# Tricarboxylic Acid (TCA) Cycle Intermediates: Regulators of Immune Responses

**DOI:** 10.3390/life11010069

**Published:** 2021-01-19

**Authors:** Inseok Choi, Hyewon Son, Jea-Hyun Baek

**Affiliations:** School of Life Science, Handong Global University, Pohang, Gyeongbuk 37554, Korea; 21600736@handong.edu (I.C.); 21500355@handong.edu (H.S.)

**Keywords:** Krebs cycle, tricarboxylic acid cycle, cellular immunity, immunometabolism

## Abstract

The tricarboxylic acid cycle (TCA) is a series of chemical reactions used in aerobic organisms to generate energy via the oxidation of acetylcoenzyme A (CoA) derived from carbohydrates, fatty acids and proteins. In the eukaryotic system, the TCA cycle occurs completely in mitochondria, while the intermediates of the TCA cycle are retained inside mitochondria due to their polarity and hydrophilicity. Under cell stress conditions, mitochondria can become disrupted and release their contents, which act as danger signals in the cytosol. Of note, the TCA cycle intermediates may also leak from dysfunctioning mitochondria and regulate cellular processes. Increasing evidence shows that the metabolites of the TCA cycle are substantially involved in the regulation of immune responses. In this review, we aimed to provide a comprehensive systematic overview of the molecular mechanisms of each TCA cycle intermediate that may play key roles in regulating cellular immunity in cell stress and discuss its implication for immune activation and suppression.

## 1. Introduction

**Figure 1:**
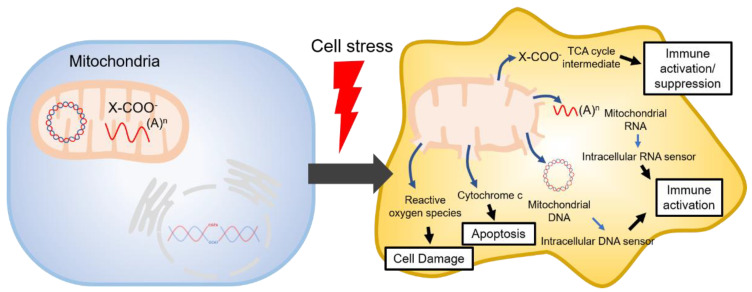
Mitochondrial danger signals in cell stress.

The tricarboxylic acid cycle (TCA, also known as the Krebs cycle or the citric acid cycle) is a series of chemical reactions used in aerobic organisms (pro- and eukaryotes) to generate energy via the oxidation of acetyl-coenzyme A (CoA) derived from carbohydrates, fatty acids and proteins. In the eukaryotic system, the TCA cycle occurs exclusively in the matrix of the mitochondria, which are surrounded by two lipid bilayers and impermeable to polar solutes. The mitochondrial membrane separates energy-producing reactions from energy-consuming ones and retains the TCA cycle intermediates, which are small, highly polar, and electrophilic organic carboxylic acids, inside mitochondria. The intermediates of the TCA cycle take several roles as metabolites in metabolisms, such as insulin secretion and fatty acid synthesis (FAS) within mitochondria. In addition, they can be transported out of mitochondria through various shuttle mechanisms to participate in various biological processes. Of note, increasing evidence suggests that the TCA cycle intermediates play crucial roles in regulating cellular immunity. In cell stress conditions, the mitochondrial membrane may be disrupted and release the TCA cycle intermediates into the cytosol, thereby impacting the cellular immunity (Figure 1).

Mitochondria are multifunctional organelles. First, they are known as the powerhouses of the cell. Mitochondria are the organelles staging the most efficient metabolic pathways, including the TCA cycle and oxidative phosphorylation supplying the entire cell with energy in form of adenosine triphosphate (ATP) [1]. In addition, mitochondria are critical in many other processes, such as detoxification, iron-sulfur cluster biogenesis, intracellular calcium homeostasis. Importantly, mitochondria are the central regulators of intracellular innate immune responses to pathogens [2,3,4,5,6,7]. In line with this, they provide stress mediators initiating cell death and autophagy, present adaptor molecules required for the transduction of cellular innate immune signals [2,3], and release mitochondrial components (e.g., mitochondrial DNA, mitochondrial RNA, cytochrome c, reactive oxygen species (ROS)) into the cytosol as danger signals [4] (Figure 1). Mitochondrial DNA and RNA may activate intracellular sensors sensing pathogenic DNA and RNA (e.g., the retinoic-acid-inducible gene (RIG)-I-like proteins, protein kinase R (PKR), the stimulator of interferon genes (STING), mitochondrial antiviral signaling protein (MAVS), cyclic guanine monophosphate (GMP)—adenosine monophosphate (AMP) synthase [cGAS] and interferon-γ-inducible protein 16 (IFI16)) to detect intracellular pathogens [2,3,8,9,10,11,12,13]. Studies have found that mitochondrial DNA leaking from dysfunctioning or disrupted mitochondria directly activates STING providing a direct potential link between mitochondrial disease and inflammatory response [3,14,15]. A more recent study showed that, under pathologic conditions, mitochondrial RNA is released from dysfunctioning mitochondria, in turn, triggering immune activation [13]. Under conditions of cell stress, mitochondria release cytochrome c and ROS inducing apoptosis and cell and tissue destruction [4]. In addition, TCA cycle intermediates may also leak from mitochondria upon cell stress and regulate cellular immune and stress responses (Figure 1).

**Figure 2:**
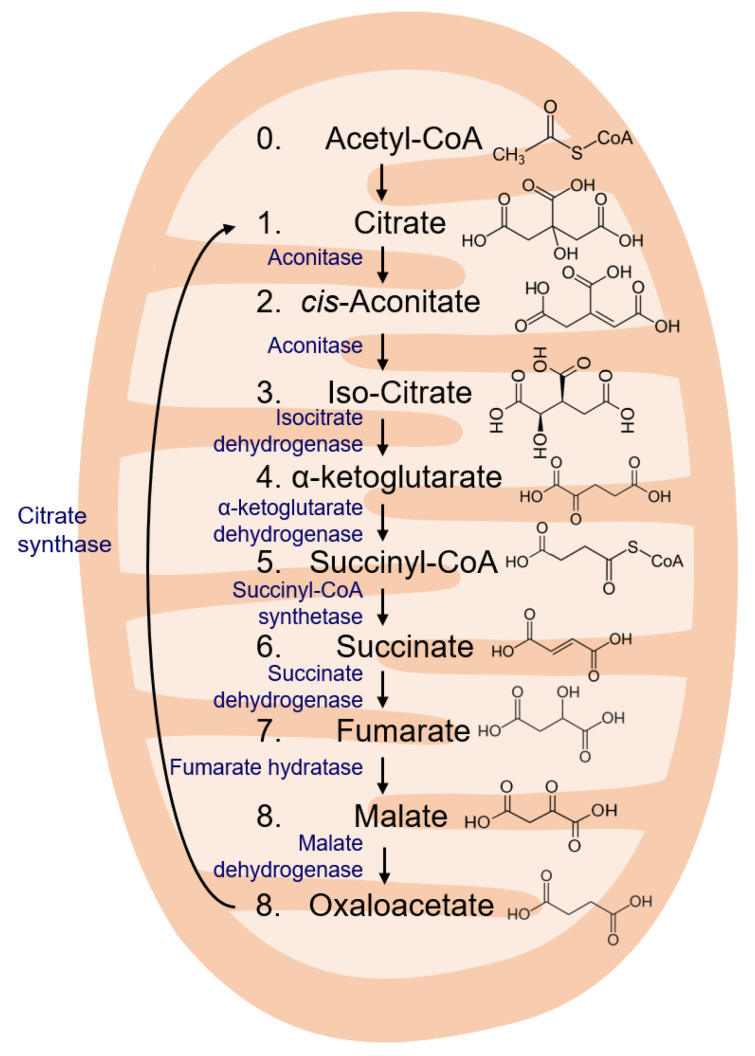
TCA cycle intermediates.

In the last ten years, it has been increasingly recognized that cellular metabolism controls immune cell functions, and it became evident that some metabolic pathways activate, and others suppress immune responses. Innovative systems biology tools have revolutionized metabolic research and are currently providing us with an overwhelming amount of data and new insights, which need to be put together to form a coherent whole. In this review, we present findings from studies focusing on the role of TCA cycle intermediates in cellular immunity and provide a comprehensive systematic overview of the immunologic features of each TCA cycle intermediate (Figure 2).

## 2. Acetyl-CoA

Acetyl-CoA is a molecule produced in the breakdown of carbohydrates, proteins, and fatty acids. It feeds into the TCA cycle by combining with oxaloacetate to citrate. Acetyl-CoA is, strictly speaking, not an intermediate of the TCA cycle, Nonetheless, the intramitochondrial levels of acetyl-CoA are essential for sustaining TCA cycle activity. Furthermore, acetyl-CoA is a precursor for fatty acids, steroids, and certain amino acids, such as glutamate, proline, and arginine [16] and has the important function of providing an acetyl group for histone acetylation in the nucleus. Elevated histone acetylation was found to upregulate the expression of glycolytic enzymes, which are related to immune activation [17]. Of note, acetyl-CoA is generated in multiple compartments, including cytosol. Therefore, it is difficult to estimate to what extent the mitochondrial portion of acetyl-CoA contributes to the expression of glycolytic enzymes. Particularly, the role of mitochondrial acetyl-CoA leaking from mitochondria during cellular stress in cellular immunity has remained elusive [18]. Interestingly, the cytosolic and nucleic levels of acetyl-CoA can increase, when citrate is transported from mitochondria into the cytosol by the dicarboxylate antiporter solute carrier family 25 (SLC25A1; mitochondrial citrate carrier, CIC) and converted into oxaloacetate and acetyl-CoA by ATP citrate lyase (ACLY). ACLY-derived cytosolic and nucleic acetyl-CoA, in turn, may drive histone acetylation affecting cellular functions [19,20,21]. The acetylation of lysine residues on histone H3 is associated with the expression of interleukin (IL)-6 in virus-infected [22] and paraquat (PQ)-treated [23] macrophages, the secretion of matrix metalloproteinase (MMP)-1 and MMP-3 in *Mycobacterium tuberculosis*–infected macrophages [24] as well as the elevated promoter activity for IL-12p40 [25]. Nevertheless, it is still unclear how histone acetylation regulates specific genes during macrophage activation. Therefore, this research area deserves further investigations in the future [26]. Acetylation can also regulate the function of proteins outside of histone modifications [27]. Specifically, acetylation of the cytoskeletal protein α-tubulin, by the tubulin acetyltransferase MEC-17, was shown to regulate IL-10 induction in lipopolysaccharide (LPS)-activated pro-inflammatory macrophages [27]. As such, acetylation can serve as an important signal to govern both pro- and anti-inflammatory cytokine production in macrophages.

## 3. TCA Cycle Intermediates

### 3.1. Citrate

Citrate is formed in the TCA cycle by the condensation of oxaloacetate and acetyl-CoA, which is catalyzed by citrate synthase. Of note, the cellular levels of citrate are increased in pro-inflammatory macrophages [28,29,30]. Mitochondrial citrate can inhibit pyruvate dehydrogenase (PDH) and succinate dehydrogenase (SDH) at high concentrations [30]. PDH converts pyruvate to acetyl-CoA and CO2, whereas SDH oxidizes succinate to fumarate in the TCA cycle. Thereby, PDH and SDH catalyze two crucial steps of the TCA cycle, of which the first is the initiation of the TCA cycle and the second the generation of FADH_2_. Overall, citrate was found to exert inhibitory effects on the TCA cycle promoting ATP-consuming pathways [30,31,32].

In pro-inflammatory macrophages, mitochondrial citrate may be transported to the cytosol by SLC25A1 and converted to acetyl-CoA by ACLY [33,34]. SLC25A1 is upregulated in pro-inflammatory macrophages in a nuclear factor κ-light-chain-enhancer of activated B cells (NF-κB) or signal transducer and transcription (STAT)-dependent manner [29,30,33,34,35]. Conversely, the inhibition of SLC25A1 leads to the reduction of inflammatory mediators [34]. In line with this, the export and breakdown of mitochondrial citrate have been linked to the production of several important pro-inflammatory mediators in macrophages, such as nitric oxide (NO), ROS, and prostaglandin E2 (PGE2) [29,30,33,35,36]. The decrease in PGE2 production may be due to a decreased availability of citrate as adding exogenous acetate rescues the effect of SLC25A1 inhibition on PGE2 production, suggesting that the cytosolic citrate is an important signal molecule in inflammation [30]. Studies have demonstrated that the upregulation of the *ACLY* gene precedes SLC25A1 activation in both resting and pro-inflammatory macrophages, although SLC25A1 might be essential for providing the substrate for ACLY activity [33,35]. It is also interesting to note that *SLC25A1* mutations that inactivate the citrate export are associated with severe mitochondrial dysfunction [29,37].

### 3.2. cis-Aconitate

*cis*-Aconitate is a TCA cycle intermediate, which is formed during the reversible transformation of citrate to isocitrate by the action of the enzyme aconitase (also known as aconitate hydratase). In this process, *cis*-aconitate normally does not dissociate from the active site of aconitase. Thus, not much is known about the cellular functions of *cis*-aconitate. Interestingly, it was recently reported that mitochondrial aconitase is associated with neurodegenerative disorders [38]. Congruently, an earlier study showed that lymphocytic mitochondrial aconitase activity is reduced in Alzheimer’s disease and mild cognitive impairment, implicating its significance in cellular homeostasis and immune cell functions [39]. In recent years, *cis*-aconitate has attracted a lot of attention because it was identified as the precursor of itaconate, which is formed from *cis*-aconitate through the decarboxylation catalyzed by the immune-responsive gene 1 (IRG1). Itaconate is upregulated upon immune activation, e.g., in response to LPS activation in macrophages, and has strong immunosuppressive properties. As the native itaconate is cell-impermeable, researchers often used structurally modified itaconate derivates with increased intracellular penetrance (e.g., dimethyl itaconate (DMI) and 4-octylitaconate) to study the intracellular functions of itaconate. So far, studies have shown that: (1) native and derivatized itaconate inhibits SDH, a component of both the TCA cycle and the Complex II of the electron transport chain (ETC) [40,41,42], consequently impairing immune activation (e.g., the hypoxia-induced factor (HIF)-1α-IL-1β axis) [41] and viral replication [42,43]; (2) activates the anti-inflammatory nuclear factor erythroid 2-related factor 2 (NRF2; also known as NFE2L2) pathway via alkylation of the Kelch-like ECH-associated protein 1 (KEAP1) protein [44,45]; and (3) regulates the translation of IκBζ and the subsequent activation of the cyclic AMP-dependent transcription factor (ATF3) albeit without affecting the primary transcriptional responses [46]. Collectively, previous studies have highlighted the immunosuppressive properties of itaconate and suggested that itaconate is an integral part of a negative-feedback loop in toll-like receptor (TLR)-mediated immune cell activation [44]. Of note, the cellular response to itaconate may differ from cell type to cell type and may also depend on the type of stimulation, as studies showed that endogenous itaconate does not induce NRF2 activation in neurons upon viral infection [42]. So far, most of the analysis on the immunosuppressive effect of itaconate has been performed in LPS-activated macrophages, and we do not know whether itaconate has the same impact on immune signaling pathways in other cell types and/or with different stimuli. Furthermore, it is to note that the most studied itaconate derivative is DMI, which is more electrophilic than the native form of itaconate. It is still unclear whether the native itaconate acts in the same way as its more electrophilic derivatives and deserves further investigations in the future.

### 3.3. Isocitrate

As mentioned above, isocitrate is formed in the TCA cycle by the isomerization of citrate catalyzed by aconitase. Studies have demonstrated an increase in citrate and a decrease in isocitrate in LPS-activated pro-inflammatory macrophages [28,33]. Furthermore, pro-inflammatory macrophages downregulate isocitrate dehydrogenase (IDH), resulting in an increased isocitrate:α-ketoglutarate ratio. Pro-inflammatory dendritic cells (DCs) and macrophages show increased glycolytic flux and a break in the TCA cycle, where pyruvate derived from glucose feeds into the TCA cycle but cannot continue beyond citrate/isocitrate. [30,47,48]. Generally, the cytosolic isoenzyme IDH1 is suppressed in cells activated by pro-inflammatory stimuli [30,49]. In glucose-deprived conditions, *IDH1* mRNA expression is increased in pro-inflammatory macrophages [30,50]. This may explain why the production of NO and ROS is decreased in glucose-deprived cells. So far, the immunological roles of cytosolic and mitochondrial iso-citrate remain elusive and deserve future investigations.

### 3.4. α-Ketoglutarate

α-Ketoglutarate is formed in the TCA cycle by oxidative decarboxylation of iso-citrate catalyzed by IDH. α-Ketoglutarate is involved in various metabolic pathways [51,52,53,54,55]. In humans, IDH exists in three isoforms: IDH1, -2, and -3. IDH1 acts in the cytoplasm and, thus, outside the context of TCA, while IDH2 and -3, as part of the TCA cycle, are found within the mitochondrial matrix [56]. α-Ketoglutarate can also be generated from glutamine and glutamate by deamination to feed into the TCA cycle [57,58], whereas α-ketoglutarate can conversely serve as a precursor of glutamine, which is a nutritionally semi-essential amino acid [59]. Previous studies have identified immunosuppressive roles for α-ketoglutarate. Similarly, L-2-hydroxyglutarate, a derivative of α-ketoglutarate, was also shown to be immunosuppressive [60,61]. In this context, it is worth noting that mutations in *IDH1* are associated with chronic inflammation [62,63]. IL-4-induced anti-inflammatory macrophages have been shown to accumulate the metabolite α-ketoglutarate [64]. It was also found that α-ketoglutarate suppresses the activation of pro-inflammatory macrophages (e.g., IL-1β expression) and supports endotoxin tolerance after activation [26,65]. α-Ketoglutarate regulates the pro-inflammatory NF-κB signaling by stimulating prolyl hydroxylase (PHD; a family of α-ketoglutarate-dependent dioxygenases (α-KGDD)). PHD1 inactivates IKK-β kinase via hydroxylation of Pro191, which, in turn, phosphorylates IκB-α, activating NF-κB [59,66]. α-Ketoglutarate inhibits stabilization of HIF-1α, a transcription factor inducing expression of immune-related genes (e.g., pro-inflammatory cytokines [67], glycolytic enzymes, and glucose transporters [68,69,70,71]) by donating an electron to HIF-specific PHD for prolyl hydroxylation leading to the degradation of HIF-1α [28,55,64,68,72,73,74,75,76]. In line with this, reduced levels of α-ketoglutarate were found to abolish PHD activity [16,55,75,77,78]. Besides, α-ketoglutarate is an important co-factor for the α-KGDD family of Jumonji-C-domain-containing histone demethylases (JMJDs) and the ten-eleven translocation (TET) proteins, which are respectively involved in histone and DNA demethylation [26,59,65,79,80,81,82,83,84,85]. Of note, histone demethylation is also associated with HIF-1α degradation [84,86]. α-Ketoglutarate facilitates macrophage polarization also via induction of fatty acid oxidation [74,87]. In addition, α-ketoglutarate produced by glutaminolysis regulates polarization of macrophages toward anti-inflammatory phenotypes, e. g., via JMJD3-mediated epigenetic changes [26,59,65,84]. Furthermore, glutamine anaplerosis (replenishing of TCA cycle intermediates from glutamate) represents an important metabolic module governing the polarization of macrophages towards an anti-inflammatory phenotype, e.g., in response to IL-4 [26,47,85,88]. Like most of the TCA cycle intermediates, α-ketoglutarate is transported from the mitochondrial matrix to the cytoplasm and vice versa. It crosses the outer mitochondrial membrane through the voltage-dependent anion channel (VDAC), and the inner mitochondrial membrane through the α-ketoglutarate/malate antiporter [59,89]. Obviously, α-ketoglutarate leaking from dysfunctioning mitochondria may exert similar anti-inflammatory functions as mitochondrial α-ketoglutarate transported into the cytosol.

### 3.5. Succinyl-CoA

Succinyl-CoA is formed by the decarboxylation of α-ketoglutarate catalyzed by α-ketoglutarate dehydrogenase (α-KGDH), a rate-limiting metabolic enzyme in the flux of the TCA cycle [78,90]. α-KGDH is responsive and sensitive to the levels of ROS impairing its function [78,91]. Of note, the increased α-KGDH activity in LPS-activated macrophages has been shown to limit the production of anti-inflammatory cytokine IL-10 [65,78]. An increase of cytosolic calcium leading to rapid mitochondrial acidification promotes α-KGDH activity, boosting NADH production and oxidative metabolism [78,92,93]. Succinyl-CoA accumulation can result in lysine succinylation of cellular proteins [28,94,95]. This post-translational modification (PTM) induces a 100-Da change in mass and masks the positive charge of the lysine side chain, likely resulting in a significant conformational change in the target protein [96]. While succinylation is largely considered to occur spontaneously, desuccinylation is at least partly enzymatic, e.g., via mitochondrial sirtuin 5 (SIRT5) [97]. LPS induces succinylation, which impacts macrophage function. LPS-induced hyper-succinylation of lysine 311 on pyruvate kinase M2 (PKM2), a key glycolytic enzyme interacting with HIF-1α and regulating aerobic glycolysis in macrophages, inhibits its pyruvate kinase activity by promoting its tetramer-to-dimer transition and induces IL-1β secretion [64,96,98,99]. LPS decreases the expression of the desuccinylase SIRT5 [28,84]. SIRT5 desucinylates various metabolic enzymes, including carbamoyl phosphate synthase 1 [84,100], SDH, PDH [84,96], acyl-CoA oxidase 1 [84,101], 3-hydroxy-3-methylglutaryl-CoA synthase 2 [84,102]. SIRT5 also desuccinylates PKM2 to prevent its entry into the nucleus and the formation of a HIF-1α/PKM2 complex [98]. Of note, the importance of this mechanism was highlighted by elevated levels of IL-1β in SIRT5-deficient mice examined in a colitis model [84,98]. Dysregulated succinate metabolism can also result in the accumulation of succinyl-CoA and lysine succinylation, a recently identified PTM [26,28,96]. Succinylation of certain proteins is found to drive immunosuppression in pro-inflammatory macrophages. Thus, the inflammatory effects of succinylation may be context-dependent and inducing various immunologic responses [28,64,103].

### 3.6. Succinate

Succinate is formed in the TCA cycle via the hydrolytic release of CoA catalyzed by succinyl-CoA synthetase. Alternatively, succinate is generated through the gamma-aminobutyric acid (GABA) shunt (a bypass of the TCA cycle, in which glutamine is used for the synthesis of glutamate, GABA, succinic semialdehyde, and eventually succinate) [28,68]. Succinate can be transported from the mitochondria to the cytosol by the dicarboxylic acid transporter. Succinate has been identified as an important metabolite in both host and microbial processes. Succinate accumulates in certain pathophysiological situations, particularly in areas of inflammation and metabolic stress [104,105]. Several studies have indicated its role of succinate in various cellular processes, such as epigenetic regulation, tumorigenesis, signal transduction, inflammation, and paracrine modulation [34,94,106,107] and have identified succinate as a metabolic signal governing local stress, tissue damage, and immunologic danger [28,108,109,110,111].

Succinate has emerged as a key player in macrophage activation [26,28,109,112]. Succinate is a highly accumulated metabolite in macrophages in response to LPS stimulation, largely depending on glutamine anaplerosis and immunometabolic reprogramming [26,28,105,112]. LPS-treated macrophages undergo a metabolic switch from oxidative phosphorylation to glycolysis, lowering the activity of the TCA cycle [113]. Of note, activated immune cells display the so-called immunologic “Warburg effect”, a term originating from cancer biology. This term describes the phenomenon that immune activation is linked to glycolysis, as cancer cells favor glycolytic metabolism [114,115], implying that immune activation requires and promotes glycolysis in a bidirectional relationship. The metabolic switch is important for the survival of immune cells at sites of inflammation, where oxygen levels are low. In this process, HIF-1α enables the cells to adapt to a low oxygen environment as a key oxygen sensor by regulating cellular responses [105,116,117]. Succinate may be accumulated following LPS treatment through SDH inhibition and post-translationally modify cellular proteins, e.g., glycolytic enzymes, including malate dehydrogenase and glyceraldehyde-3-phosphate dehydrogenase (GAPDH) [28,88,117,118]. Notably, a high succinate/α-ketoglutarate ratio was linked to the pro-inflammatory phenotype, whereas a low ratio was associated with macrophage polarization towards an anti-inflammatory phenotype [65,84]. As PHD dehydroxylation converts oxygen and α-ketoglutarate to succinate and CO_2_, high levels of succinate can slow PHDs through product inhibition [105,119]. Importantly, the balance between succinate and α-ketoglutarate levels intracellularly regulate members of α-KGDDs involved in epigenome remodeling and innate immune memory [26,59,65,120]. Like PHDs, α-KGDDs undergo competitive inhibition by succinate, and so the ratio of α-ketoglutarate to succinate can often be a key determinant of their enzymatic activity [26,59]. LPS-induced succinate can enhance the pro-inflammatory activity in macrophages by promoting the expression of the key pro-inflammatory cytokine IL-1β [28,113,117,121,122,123]. In effect, succinate behaves as a danger signal or alarmin to sustain the production of IL-1β by promoting the binding of HIF-1α to a hypoxia-response element (HRE) in its promoter [26,28,84,110,113,117,123,124,125]. HIF-1α down-regulates oxidative phosphorylation by inducing PDH kinase 1 (PDK1) and 3 (PDK3), both of which in turn also inactivate PHD, allowing for further stabilization of HIF-1α [88,126,127,128,129]. It has been demonstrated that pharmacologic inhibition of SDH or RNA interference of subunit B of SDH, induces HIF-1α stabilization in a ROS-dependent manner [34,130]. More recently, succinate oxidation in LPS-activated macrophages was shown to inhibit PHD activity indirectly via mitochondrial ROS resulting in HIF-1α stabilization and IL-1β production [26,112]. This effect is blocked by α-ketoglutarate, the substrate for PHD that generates succinate as a by-product in HIF-1α hydroxylation [28,131]. Succinate also undergoes oxidation by SDH to drive mitochondrial ROS generation from the Complex I [26,28,112]. Consequently, mitochondrial ROS production increases, subsequently stabilizing HIF-1α, the driver of additional pro-inflammatory responses in macrophages [28,64,112,132].

In DCs, succinate triggers intracellular calcium mobilization, induces migratory responses, and acts in synergy with TLR ligands to induce the production of pro-inflammatory cytokines [84,111]. Indeed, IL-1β expression is enhanced in murine bone marrow-derived DCs treated with succinate and LPS simultaneously [111,117]. Succinate was found to act in synergy with the TLR3 ligand poly(I:C) and the TLR7 ligand imiquimod, increasing TNF-α production [34,133]. Succinate also increases the capacity of DCs to present antigens to T cells and induce adaptive immune responses, which may further exacerbate inflammation [117,134]. When DCs are primed with succinate and antigen simultaneously, antigen-specific T cell activation is elevated, as measured by increased tumor necrosis factor (TNF)-α and interferon (IFN)-γ production from these cells [117,135]. Interestingly, HIF-1α-deficient DCs show impaired ability to activate T cells [136], suggesting that this transcription factor is important for T cell polarization and activation [34]. In the adaptive compartment, HIF-1α activation favors the differentiation of T lymphocytes into pro-inflammatory Th17 cells and attenuates regulatory T (Treg) cell development [34,137]. An increase in succinate caused by a loss of Complex III was also shown in Tregs to inhibit TET activity, which is necessary to maintain immune regulatory gene expression and suppressive function [60,84]. Succinate can induce the activation of hematopoietic stem cells, and regulate their proliferation, migration, and apoptosis *in vitro*, along with increasing the secretion of inflammatory cytokines, such as IL-6 and TNF-α [94,138,139]. Succinate also accumulates in hypoxia during ischemia-reperfusion injury, where its accumulation and oxidation control ROS production and contributed to the severity of tissue injury [84,110,140,141]. Similarly, succinate is also associated with inflammatory diseases [64,142]. Elevated amounts of circulating succinate occur in some physiological conditions, such as endurance exercise [109,143] and in certain pathologies, including inflammatory bowel disease [144,145,146,147,148,149,150,151,152,153], sepsis [28,60,154], hypertension, ischemic heart disease [109,155] type 2 diabetes mellitus [109,156,157] and obesity [109,156,158]. Numerous studies have identified tissue-specific and systemic effects of succinate as a pro-inflammatory mediator [105,106,111,157]. During inflammation, IL-37 restores cellular metabolism by reducing succinate, inhibiting mammalian target of rapamycin (mTOR), and activating AMP-activated protein kinase (AMPK) [159]. The role of succinate as a signaling molecule in inflammation and disease has been discussed in more detail elsewhere [28,34,160].

As succinate is not membrane-penetrant, researchers generated a succinate derivative which is cell-permeable. Using diethyl succinate, they increased the intracellular levels of succinate [64]. However, diethyl succinate appears to be immunosuppressive similar to DMI and dimethyl fumarate (DMF), suppressing LPS-induced pro-inflammatory cytokine secretion, gene and marker expression (such as IL-1β, IL-6, TNF-α, NO, CD40, CD86) in bone marrow-derived macrophages [64]. Another cell-permeable derivate of 4C organic compound, which was used to study the intracellular function of succinate was dimethyl malonate (DMM). DMM is an inhibitor for SDH, which increases intracellular levels of succinate in macrophages [64,112]. Here again, DMM showed a dose-dependent inhibition of inflammatory mediators IL-6, TNF-α, and NO. This may suggest a role for intracellular succinate to inhibit inflammatory responses, as SDH activity is described to be altered in pro-inflammatory macrophages, thus leading to succinate accumulation. We note that derivatized TCA cycle intermediates generally exhibit strong immunosuppressive features. The derivatization of TCA cycle intermediates may drastically increase the electrophilicity of the molecule, and the native and derivative may act via different chemical reactions [46,161,162]. Overall, studies have shown that endogenous succinate promotes immune activation. Obviously, succinate leaking from dysfunctioning mitochondria may exert similar pro-inflammatory functions as mitochondrial succinate transported into the cytosol.

### 3.7. Fumarate

Fumarate is formed in the TCA cycle by the SDH-catalyzed dehydrogenation of succinate [104,117,161,163]. Activation of innate immune cells, such as macrophages and monocytes, with pro-inflammatory stimuli (e.g., LPS, IFN-γ) leads to remodeling of the TCA cycle and intracellular accumulation of fumarate [28,41,124,164,165,166]. LPS-activated macrophages show a substantial increase in flux through the induction of aspartate-arginosuccinate shunt, which fuels and maintains the TCA cycle flux [47,165]. Furthermore, being connected to the TCA cycle at fumarate, the induced aspartate-arginosuccinate shunt causes the accumulation of fumarate in activated macrophages [165]. Interestingly, the inhibition of the aspartate-arginosuccinate shunt leads to the downregulation of the pro-inflammatory mediators, including NO and IL-6, along with suppression of the ‘immunologic Warburg effect’ in activated macrophages [165]. Therefore, replenishing fumarate seems to be important for metabolic reprogramming of macrophages during inflammation [165]. The elevated cytokine response in these cells is linked to an increase in the amount of fumarate sufficient to drive the response by activating HIF-1α [33,167]. The accumulation of fumarate by inhibiting fumarate hydratase (FH; also known as fumarase) with FH inhibitor 1 (FHIN1) impairs cell death and reduces the formation of gasdermin D, the executioner of pyroptosis [164]. The in vivo-compatible FHIN2 elevates FH levels in vivo, while reducing IL-1β, indicating that fumarate inhibits pyroptosis in vivo [164]. Loss of function of FH in cancers increases fumarate levels leading to elevated accumulation of NRF2 [168,169]. Fumarate can attenuate autoimmune diseases, in part, through modulation of DC maturation and antigen-presenting capacity [170,171,172,173]. In general, fumarate is considered anti-inflammatory. Like other TCA cycle metabolites, fumarate is a cell-impermeable polar molecule. Therefore, researchers developed fumarate derivatives with increased intracellular penetrance to study immunological features of fumarate. Of note, the stable derivative of fumarate (DMF, trade name: Tecfidera^®^), is known to be a strong immunosuppressive. DMF is a leading oral therapy for multiple sclerosis. DMF is more electrophilic than fumarate and other structurally similar metabolites, such as DMI, and itaconate [46]. Therefore, whether the native fumarate has a similar effect in vivo and in vitro has yet to be determined [161,162]. Whereas the precise mechanism of immunosuppression has remained elusive, increasing evidence indicates that the critical mechanism of DMF is related to its high electrophilicity, which allows DMF to modify proteins by reacting with cysteines via an electrophilic (Michael-type) addition [174,175], impacting the function of proteins. Several groups have proposed that DMF stimulates an antioxidant response by modifying cysteine residues in the NRF2-KEAP1 complex, which is a major electrophile response pathway in mammals [161,168,176,177,178,179,180]. The transcription factor NRF2 has been shown to alleviate inflammation by inducing heme oxygenase-1 (HO-1), which potentially increases IRG1 and downregulates TNF-α expression [180,181,182,183,184]. An NRF2 deficiency causes an exacerbation of inflammation in a variety of murine models, such as sepsis, pleurisy, and emphysema [185,186,187,188], as well as autoimmune phenotypes in some murine strains [189,190]. GAPDH has been hypothesized to play a key role in the mechanism of action of DMF [191]. DMF has been suggested to modify the active site cysteine (Cys152 in humans) and to inactivate GAPDH. Thereby, DMF may downregulate glycolysis, ultimately causing immunosuppression [191]. However, a recent study suggested that this mechanism does not have the primary role in DMF-mediated immunosuppression [164].

Nonetheless, the exact role of intracellular fumarate during inflammation is yet to be investigated [165]. Outside mitochondria, succinate and fumarate can inhibit certain demethylation reactions. Accumulation of succinate and fumarate leads to the inhibition of DNA demethylases and lysine demethylases (KDMs) via product inhibition. Accordingly, fumarate accumulation (e.g., derived from glutaminolysis) was shown to inhibit KDM5 histone demethylases, altering histone H3 at lysine 4 (H3K4) methylation at the promoters of pro-inflammatory cytokines, such as *Tnfa* and *Il6* [33,85,86,161,167,192,193,194,195,196] and regulating the chromatin-modifying activities of histone acetyltransferases or deacetylases by altering their post-translational lysine methylation status [194]. Fumarate induces epigenetic changes in macrophages that are associated with trained immunity [86,193]. Interestingly, exposing monocytes to fumarate, but not succinate or malate, for 24 h recapitulated the trained macrophage phenotype in terms of augmented pro-inflammatory cytokine production capacity. This was accompanied by an enrichment of H3K4me3 on the promoters of the cytokine genes, due to a direct inhibitory effect of fumarate on the KDM5 family of histone demethylases, which are responsible for demethylation of H3K4 [86,194,197]. This effect could partly be restored by the addition of α-ketoglutarate [194,195,196,197]. The striking elevation of succinate and fumarate induced by the metabolic rewiring of trained macrophages, therefore, represents a plausible mechanism underlying the integration of immunometabolic and epigenetic programs in trained immunity [82,194]. Fumarate degradation is detrimental to the host and abrogates trained immunity, promoting recurrent infections [193].

### 3.8. Malate and Oxaloacetate

L-Malate is formed by the hydration of fumarate catalyzed by FH. In the last reaction of the TCA cycle, L-malate dehydrogenase oxidizes L-malate to oxaloacetate. Not much is known about the immunological role of intracellular L-malate and oxaloacetate, although the activation of macrophages with LPS results in increased levels of L-malate [28,67]. Oxaloacetate serves as a strong inhibitor of SDH, thereby limiting post-ischemic oxidation of succinate. It would be worth examining whether the endogenously formed oxaloacetate conveys protection through reduced SDH activity during reperfusion [198]. Future studies are needed to determine the immunological roles of L-malate and oxaloacetate during a mitochondrial disruption.

## 4. Discussion

**Figure 3:**
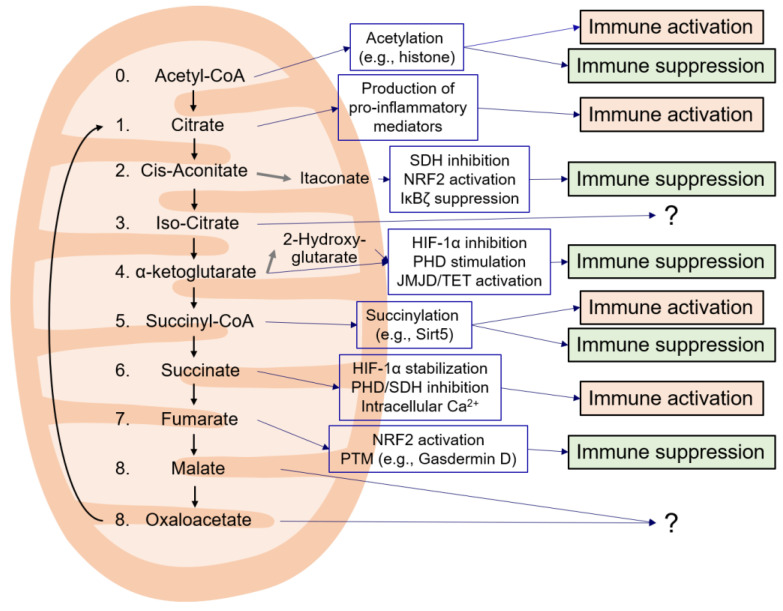
Immunological features of the TCA cycle intermediates. **?**: Unknown function.

This review has provided insights into the immunological roles of TCA cycle intermediates that may leak from dysfunctioning mitochondria along with other mitochondrial danger molecules and regulate the cellular immunity. In cells with mitochondrial stress, TCA cycle metabolites, such as citrate, itaconate, succinate, fumarate, L-malate may accumulate despite decreased mitochondrial respiration and link cellular metabolism to innate leukocyte responses [28,64,67,124,161,165,166,199]. Activation of immune cells leads to the remodeling of the TCA cycle via a process called the “immunologic Warburg effect”. The TCA cycle intermediates citrate, α-ketoglutarate, succinate, and fumarate regulate inflammatory gene expression [26,33,85,86,161,167,192,193,194,195,196]. Innate immune cells use ROS as a key signaling and functional molecule during inflammation. TCA cycle intermediates such as succinate, citrate, fumarate, and itaconate are thereby important regulators of the production of ROS [167]. Acetyl-CoA, succinate, and fumarate may also regulate the innate immune responses by post-translationally modifying proteins via acetylation, succinylation, and succination [85,200]. Certain metabolites such as acetyl-CoA, succinate, NAD^+^, and α-ketoglutarate can serve as co-factors for epigenetic enzymes, thus potentiating innate immune memory [36,196]. It is important to note the balancing of succinate, α-ketoglutarate, and fumarate levels in macrophages can alter the epigenetic landscape of the cell, profoundly impacting macrophage polarization and innate immune memory [26,82,161,201,202]. Overall, studies have shown that citrate and succinate have pro-inflammatory properties, whereas itaconate, α-ketoglutarate, and fumarate are linked more with immunosuppressive roles (Figure 3). Interestingly, most of the latter metabolites are upregulated during immune activation, indicating that they balance immune activation and suppression. The immunological roles of some TCA cycle metabolites, such as iso-citrate, L-malate, oxaloacetate, are only partially understood and deserve further investigation in the future (Figure 3).
